# Anesthesia and Perioperative Management for Surgical Correction of Neuromuscular Scoliosis in Children: A Narrative Review

**DOI:** 10.3390/jcm12113651

**Published:** 2023-05-24

**Authors:** Jan Hudec, Tereza Prokopová, Martina Kosinová, Roman Gál

**Affiliations:** 1 Department of Anesthesiology and Intensive Care Medicine, Faculty of Medicine, Masaryk University, University Hospital Brno, 601 77 Brno, Czech Republic; hudec.jan@fnbrno.cz (J.H.); prokopova.tereza@gmail.com (T.P.);; 2 Department of Simulation Medicine, Faculty of Medicine, Masaryk University, 625 00 Brno, Czech Republic; 3 Department of Pediatric Anesthesiology and Intensive Care Medicine, Faculty of Medicine, Masaryk University, University Hospital Brno, 625 00 Brno, Czech Republic

**Keywords:** neuromuscular scoliosis, anesthesia, total intravenous anesthesia, children, spondylosurgery, spine

## Abstract

Scoliosis is the most frequent spinal deformity in children. It is defined as a spine deviation of more than 10° in the frontal plane. Neuromuscular scoliosis is associated with a heterogeneous spectrum of muscular or neurological symptoms. Anesthesia and surgery for neuromuscular scoliosis have a higher risk of perioperative complications than for idiopathic scoliosis. However, patients and their relatives report improved quality of life after the surgery. The challenges for the anesthetic team result from the specifics of the anesthesia, the scoliosis surgery itself, or factors associated with neuromuscular disorders. This article includes details of preanesthetic evaluation, intraoperative management, and postoperative care in the intensive care unit from an anesthetic view. In summary, adequate care for patients who have neuromuscular scoliosis requires interdisciplinary cooperation. This comprehensive review covers information about the perioperative management of neuromuscular scoliosis for all healthcare providers who take care of these patients during the perioperative period, with an emphasis on anesthesia management.

## 1. Introduction

Scoliosis is the most common spinal deformity in children. It is an abnormal lateral deviation of the spine, greater than 10° in the frontal plane. The lateral curvature is typically in combination with deviation in the sagittal plane (hyper/hypokyphosis or hyper/hypolordosis) and vertebral rotation [1,2]. The pathophysiological effect of scoliosis is complex and multiorgan. Besides musculoskeletal disturbances, cardiovascular, pulmonary, or psychosocial effects are described [1,2,3,4]. Scoliosis is categorized according to the etiology. The most frequent types include idiopathic (80%), congenital, and neuromuscular scoliosis [1,3]. Neuromuscular scoliosis, scoliosis associated with any neuromuscular syndrome, can be followed by alterations of vital functions by underlying diseases. Patients are at higher risk of curve progression to a significant deviation, which can lead to ventilation/perfusion (V/Q) mismatching, respiratory failure, and cor pulmonale [3,5,6,7]. The surgical correction of any scoliosis is indicated to prevent curve progression or the progression of restrictive lung disease. Other indications, especially in neuromuscular scoliosis, include improving posture and nursing care. Although there is a higher rate of perioperative complications in neuromuscular scoliosis surgery, patients and their relatives report improvements in quality of life after the surgery [7,8,9].

Scoliosis surgery is a major surgery associated with a high possibility of serious complications, particularly in patients with neuromuscular scoliosis. According to our experience, the management of patients undergoing surgery for idiopathic scoliosis is well described in many articles. This narrative review is focused on the differences and specifics of pediatric patients with neuromuscular scoliosis. Our goal is to bring relevant and comprehensive information to all perioperative team members, such as anesthesiologists, pediatricians, surgeons, or neurophysiologists, since interdisciplinary cooperation could be crucial for the patient outcome. We highlight the challenges both in the perioperative management of scoliosis surgery itself and in high-risk anesthesia for patients with neuromuscular disease [9,10,11].

## 2. Methods

The main limitation for the research was the diversity and rareness of some neuromuscular syndromes and the fact that neuromuscular scoliosis correction is a serious surgery but also a marginal topic requiring specialized care. For this reason, we choose an extended time frame of 1995–2022. The Google Scholar (https://scholar.google.com, Access time: 1–31 August 2022) [12], PubMed (https://pubmed.ncbi.nlm.nih.gov/, Access time: 1–31 August 2022) [13], Embase (https://www.embase.com/, Access time: 1–31 August 2022 [14] and Web of Science databases (https://www.webofscience.com/wos/author/search, Access time: 1–31 August 2022) [15] were used to search the literature. The aim was to map the whole perioperative process (preanesthetic assessment, anesthesia, and postoperative care).

We used a combination of terms, such as “anesthesia”, “anesthetic management”, “neuromuscular disease”, neuromuscular disorders”, “neuromuscular scoliosis”, “pediatric”. Besides this, we focus on specific issues in each section of this article.

Preanesthetic assessment Medical Subject Headings (MeSH) terms: “airway management”, “respiratory functions examination”, “cardiovascular examination”, “neurological examination”, invasive access”, “nutrition”, “prehabilitation”.

Anesthesia and intraoperative MeSH terms: “air embolism”, “blood management”, “intraoperative neurophysiological monitoring”, “malignant hyperthermia”, “orphananesthesia”, “perioperative monitoring”, “prone position”, “rhabdomyolysis”, “temperature management”, “total intravenous anesthesia”, “vital functions monitoring”.

Postoperative care MeSH terms: “intensive care unit”, “postoperative care”, “respiratory insufficiency”, “ventilator weaning”, “hemodynamic monitoring”, “emergence delirium”, “pain management”, “postoperative pain”, “fluid therapy”, “acute kidney injury”, “nutritional support”, “postoperative hemorrhage”, “postoperative complication”, “postoperative wound infection”, “rehabilitation”.

## 3. Results

### 3.1. Preanesthetic Assessment

The preanesthetic evaluation requires a multidisciplinary approach. An integral part of this assessment is the identification of decompensated functions or organ reserves that could bring possible perioperative complications and its maximal preoperative optimization [9,10]. Anesthesiologists should individually consider the extent of preoperative testing according to the risks and benefits, scoliotic curvature severity, mental state or physical status with comorbidities, and type of planned surgical technique. The administration of pharmaceutical premedication has to be considered individually in patients with neuromuscular disorders. There are no precise data about dose restriction; it should be decided with respect to the neurological status and other associated aspects, such as the vagus stimulator, Lioresal pump, or severity of lung disease [10,16,17,18]. We list the specifics of preanesthetic assessment divided according to the ABCDE approach. This represents a widely respected approach to seriously ill patients, including the pediatric population. This approach could be used to anticipate and systematically prevent life-threatening complications [19].

#### 3.1.1. Airways

Difficult airway management should be expected with a higher rate in patients with neuromuscular disorders as some syndromes are associated with facial dysmorphism, abnormal habitus, or limited mobility, especially limited neck mobility [17,19]. Anesthesiologists should search for risk factors for difficult airway management during the preanesthesia visit and prepare for airway securing, including advanced airway techniques such as video laryngoscopy or awake fiber optic intubation [20,21,22].

#### 3.1.2. Breathing

The evaluation of respiratory function should be extended to chest X-radiation (X-ray), peripheral capillary oxygen saturation (SpO_2_) measurement, and pulmonary function tests such as spirometry. Chest X-ray can provide information about airway deviations or signs of recurrent gastric content aspiration [10,18]. Spirometry quantifies the type and severity of lung dysfunction, typically restrictive lung disease. However, a development delay may impair the feasibility of spirometry. Severe restrictive lung disease (forced vital capacity <50% of normal values in patients with muscle weakness and symptoms of hypoventilation, or forced vital capacity <30% of normal values without muscle weakness) predicts a higher risk of respiratory complications such as pneumonia or prolonged weaning from ventilatory support [10,23]. Another method to determine high-risk patients (when spirometry is not available) is carbon dioxide monitoring during sleeping. However, this method is unreliable to detect hypoventilation and it is not preferred nowadays. Non-invasive bioelectrical impedance is available for clinicians. These monitors measure the tidal volume, minute ventilation, or respiratory rate to detect hypoventilation. It is essential to reduce the risk of perioperative complications and respiratory failure by optimizing respiratory function. Prehabilitation in cooperation with physiotherapists and pneumologists can improve patients’ outcomes. This prehabilitation includes preoperative training in non-invasive ventilation or assisted coughing [23,24,25].

#### 3.1.3. Circulation

Electrocardiography (ECG) is a standard part of preoperative assessment that can detect arrhythmias potentially associated with autonomic dysfunction [9,10,11]. Careful cardiologist examination with echocardiography is recommended in patients with myocardial dysfunction, because many neuromuscular syndromes are associated with heart disease, such as cardiomyopathy, valve disease, congenital heart disease, or limited stress tolerance. Preoperative cardiologic evaluation should be suggested in patients without myocardial dysfunction if pulmonary hypertension is suspected [10,20,26,27].

#### 3.1.4. Disability (Neurology)

The neurological examination must provide a precise diagnosis with the known pathophysiology of a neuromuscular disorder. It helps with individualized anesthesia planning, which should reduce the risk of many anesthetic complications (see below). Exact neurological status description is suitable for juridical reasons, in case of postoperative neurological deficit development [27]. In addition, neurological deficit description helps with physiotherapy planning [26,27]. Epilepsy is diagnosed more frequently in patients with neuromuscular disorders [28]. Actual electroencephalography (EEG) should be considered, and perioperative antiepileptic drug administration must respect neurologist recommendations, taking into account possible interactions with anesthetic drugs [26,27].

#### 3.1.5. Exposure (Environment)

Signs of difficult invasive access and suitable places for cannulation should be actively searched for. The signs of difficult access include muscle contractures or abnormal body proportions [27]. Other specific examinations should be indicated individually, e.g., nutritionists’ recommendations in patients with signs of malnutrition [9,29]. Except for standard blood tests, plasma levels of myoglobin or creatine kinase should be obtained. These markers are often altered in patients with neuromuscular disease. However, plasma levels before surgery are helpful to determine the baseline and monitor these levels’ dynamics after surgery when suspecting rhabdomyolysis development [27,30,31].

### 3.2. Anesthesia and Intraoperative Management

Anesthesia for neuromuscular scoliosis is a challenge for the anesthetic team. Intraoperative management is specific and unique due to neuromuscular disease in children with its possible associated complications, such as intraoperative neurophysiological monitoring (IONM), high blood loss, patient positioning—typically pronation—fluid shift, long surgical times, and body temperature loss [10,27,31].

#### 3.2.1. Anesthetic Management

Anesthesia and the choice of anesthetic drugs have to respect the pathophysiological category of the syndrome. Data on orphan syndromes are scarce. However, anesthesiologists can obtain information about the syndromes and anesthetic management from internet sources, e.g., published unique case reports, Orphananesthesia [32]. Total intravenous anesthesia (TIVA), most often the combination of propofol and remifentanil, is the most suitable and safe method, especially in the case of IONM. It requires a sufficient intravenous (IV) line. However, cannulation can be difficult due the joint contractures or abnormal habitus [33,34]. The indication of a central venous catheter should be considered individually according to the comorbidities and expectation of the need for vasopressors [10,27]. Ultrasound-guided cannulation can reduce the incidence of unsuccessful attempts and also increases the safety of the procedure [10,20]. Depth of anesthesia monitoring is recommended for TIVA to prevent overdosing and shorten the time of awakening after the surgery [10,18,27].

Non-depolarizing muscle relaxants can be administered safely. The effect of these drugs is variable, typically prolonged in patients suffering from neuromuscular disorders. Therefore, any administration of neuromuscular blocking agents should be followed by monitoring of the depth of neuromuscular blockade [26,27,32]. Rocuronium with available antagonist sugammadex represents a safe combination to provide complete recovery after the blockade and allow reliable IONM [35].

Anesthesia and vital functions should be managed individually with consideration of the age and comorbidities of each patient. The aim of ventilation and oxygenation is normoxia and normocapnia [9,10]. Anesthesiologists can manage perioperative vital functions according to vital function levels mentioned in the European Pediatric Advanced Life Support (EPALS) Guidelines 2021 [36]. The target blood pressure is not strongly recommended in children during scoliosis surgery. However, some data recommend maintaining a mean arterial blood pressure (MAP) near 65 mmHg, but approximately the fifth percentile of the mean and systolic arterial blood pressure for the relevant age group can be used safely. Transesophageal echocardiography should be considered in high-risk patients, e.g., patients with myocardial dysfunction or pulmonary hypertension. In addition, intraoperative transesophageal echocardiography helps to evaluate the volume status and manage goal-directed fluid therapy [10,33,37].

#### 3.2.2. Neuromuscular Disease and Associated Complications—Malignant Hyperthermia and Rhabdomyolysis

Perioperative complications in patients with neuromuscular syndrome are observed at a higher rate compared to patients without neuromuscular disorders [9]. The most severe complications are malignant hyperthermia (MH) and rhabdomyolysis. Their development depends on the pathophysiology category of concrete neuromuscular disease, e.g., myopathies represent a high risk for MH or rhabdomyolysis [38].

MH is a syndrome caused by the hypermetabolic response with increased carbon dioxide production to suxamethonium or volatile anesthetics exposure. Early diagnosis and treatment with cooling of the patient are essential. It is recommended to use local protocols (personalized guidelines with particular locations of, e.g., dantrolene and emergency numbers) for the treatment of malignant hyperthermia. These protocols should be available in the operation room, where surgeries for high-risk patients are performed. To manage the crisis, the elimination of triggers (mentioned anesthetic drugs), hyperventilation with 100% oxygen, treatment of hyperkalemia (calcium administration for membrane stabilization, salbutamol, and insulin/glucose administration for kalemia reduction) are necessary. Dantrolene administration and symptomatic therapy, such as arrhythmia treatment, should be managed according to the actual guidelines [38,39,40].

Rhabdomyolysis is a condition associated with muscle damage. Myoglobin, potassium, and creatine kinase are released from the intracellular space [41]. Rhabdomyolysis was described after succinylcholine or volatile anesthetics exposure as anesthesia-induced rhabdomyolysis. Moreover, it was reported after muscle injury during a long surgery with inadequate patient positioning [38,42]. The differential diagnosis of MH can be complicated, and both syndromes can initially present with similar clinical signs. Rhabdomyolysis is typical of hyperkalemia, creatine kinase elevation, or myoglobinuria with “cola-colored urine”. Myoglobinuria can be monitored postoperatively. Therapy has to focus on hyperkalemia treatment, hyperventilation with 100% oxygen, the prevention of acute kidney injury development, volume therapy, and the elimination of triggers [38,41,42].

#### 3.2.3. Intraoperative Neurophysiological Monitoring

IONM represents an important and, in recent years, rising method for neural structure injury monitoring. Anesthetic management, including oxygenation, ventilation, massive blood loss, or hypotension, can influence the IONM reproducibility. IONM reproducibility can be limited in patients with neuromuscular disorders. The anesthetic team has to create the best conditions for IONM. The most common anesthetic technique is TIVA, typically in a combination of propofol and remifentanil. This combination is suitable for patients with neuromuscular disorders and allows early awakening from anesthesia if surgeons require the wake-up test. Other intravenous agents can be applied considering their adverse effect profiles and contraindications. Modern trends show the use of ketamine in combination as a co-analgesic, which reduces the total dose of propofol and remifentanil. All neuromuscular relaxants interfere with motor evoked potential (MEP) monitoring. They are administered in the phase of induction to the anesthesia to facilitate airway securing. Other anesthesiological aspects include maintaining normoxia with normocapnia, preventing severe hypothermia (more than 2.5 °C from the baseline), and ensuring adequate blood flow to the spinal cord. This means maintaining normotension during surgery (see above), namely increasing MAP above 85 mmHg when MEP is decreased. Anesthesiologists must consider the transfusion trigger to ensure adequate perfusion and oxygenation (see below) [43,44].

#### 3.2.4. Positioning and Associated Complications

The prone position is the most frequent position for scoliosis surgery to facilitate access to the spine. This position is associated with several complications caused by raised intra-abdominal or thoracic pressure. In addition, prone positioning is associated with a higher risk of postoperative visual loss [45,46,47,48].

Patients with neuromuscular disorders can suffer from joint and muscle contractures, and some syndromes can be associated with low bone density. There is a potential risk of iatrogenic injury. These conditions represent increased demands for exact positioning. Correct but considerate prone positioning, respecting joint mobility, and the pressure distribution on the chest and pelvis decrease the rate of complications. Aside from careful positioning on the operating room table, the aesthesia team should elevate the upper part of the body to decrease the intraocular pressure and reduce the risk of visual loss. Other factors in reducing the risk of postoperative visual loss are avoiding anemia, hypotension (see above), and preventing venous return obstruction due to malpositioning. Besides ensuring appropriate positioning before surgery, the anesthetic team should control the body position during surgery. The patient may be harmed by manipulation by the operating team during surgery [45,46].

Advanced cardiopulmonary resuscitation with chest compressions is possible in a prone position. External cardiac massage may be performed with the palms placed over the patient’s scapulae. Internal cardiac massage and direct defibrillation were performed successfully via left posterior thoracotomy in a patient with Duchenne’s muscular dystrophy during scoliosis correction in a prone position [48]. However, anesthesiologists should consider turning the patient to a supine position in the case of a stabilized spine without prominent orthopedic tools for manipulation with instrumentation (rod pushers, connect ratchets, downtubes, etc.) [10,47,48].

#### 3.2.5. Blood Loss and Patient Blood Management

Patients with neuromuscular disease are at a high risk of extensive blood loss because of prolonged and extensive procedures or bones with lower density. Strategies to reduce blood loss are still being discussed. Antifibrinolytics’ effect in reducing blood loss and transfusion administration has been described in scoliosis surgery, especially in patients with neuromuscular disorders. Tranexamic acid is one of the most widely used antifibrinolytics. The recommended prophylactic dose is about 15 mg/kg IV. The optimal maintenance dose is unclear; current data mention infusions of 1–20 mg/kg/h. The risk of adverse events, thromboembolism included, has not been increased in prophylactic administration during scoliosis surgery [49,50,51].

Another means to reduce allogeneic blood transfusion is intraoperative cell salvage (Cell Saver). The blood is collected from the wound into a reservoir. Red blood cells can be re-infused to the patient after purification of the collected blood. It is a preferred method, especially in high-risk patients with neuromuscular disorders and a presumed low bone density [52,53].

Desmopressin increases von Willebrand factor and factor VIII levels. However, it does not have an effect in reducing blood loss. Prophylactic administration is not recommended [54].

Protocols for red blood cell transfusion triggers describe the administration of Red Blood Cells (RBC) at levels between 7 and 8 g Hb/dL. Other coagulation factors should be administered according to the laboratory or viscoelastic hemostatic assays ideally [54,55].

Patients with neuromuscular disorders or immobilization after extensive surgery, in combination with a post-surgical inflammatory state, are at a higher risk of deep vein thrombosis and pulmonary embolism. Early mobilization and mechanical prevention are recommended. Data on pharmacological prophylaxis are scarce. Low-molecular-weight heparin administration in the perioperative period until the normalization of the patient’s condition should be considered after the validation of risk factors [56].

#### 3.2.6. Body Temperature Management

As mentioned above, severe hypothermia, with a decrease in temperature of more than 2.5 °C from the baseline, interferes with IONM. Patients with neuromuscular disease, a lower body mass index, or a larger Cobb angle (the angle between the extension line of the upper and lower end plate of the most inclined vertebral bodies) are at a higher risk of hypothermia [5]. Other adverse effects include prolonged metabolism of anesthetic agents, coagulopathy with higher blood loss, and wound or respiratory infections. Preoperative and intraoperative active warming, or prewarming before surgery, is recommended to prevent hypothermia in patients with neuromuscular disorders [57,58].

### 3.3. Postoperative Care

#### 3.3.1. Admission to Intensive Care Unit (ICU)

According to studies, most of the patients require admission to the ICU or pediatric ICU. However, the decision should be made individually, e.g., the recovery after surgeries with a duration under 4 h can be, in some cases, manageable at a postanesthesia care unit. The possible predicting factors for ICU admission are a low weight, neuromuscular scoliosis, pre-existing respiratory pathology, and other comorbidities. A higher number of operated segments or a higher degree of spine curvature is not associated with ICU admission [59,60].

#### 3.3.2. Airway and Breathing

Patients with neuromuscular scoliosis often suffer from preexisting respiratory diseases such as muscle weakness (e.g., Duchenne’s muscular dystrophy) or restrictive disorders, most often caused by chest deformities, which leads to a risk of postoperative respiratory failure [61,62]. Facial dysmorphism in some syndromes is associated with difficult airways. Proper equipment for difficult airway management should be available at the ICU in case of the need for emergent reintubation. Reintubation or prolonged weaning is associated with a risk of ventilator-associated pneumonia or tracheal stenosis, so early successful weaning should be one of the main goals [63]. Bulbar weakness can also be part of some syndromes and lead to dysphagia, gastric regurgitation, dysphonia, or difficult expectoration [62]. Protocols for weaning and monitoring diaphragm function can help to achieve early extubation, leading to better patient outcomes [64,65]. Implementing non-invasive ventilation into weaning processes in high-risk patients can prevent prolonged intubation and tracheostomy [62,66,67,68].

#### 3.3.3. Circulation

Early after the operation, at least ECG, non-invasive blood pressure, and peripheral oxygen saturation should be monitored. For patients without signs of hemodynamic instability (hypotension, arrhythmias, altered mental status, low diuresis, etc.), standard monitoring is sufficient. In cases of hemodynamically unstable patients, invasive blood pressure should be monitored. Hypotension should be treated with volumotherapy and vasopressors according to its severity. For the prediction of volume responsiveness in hemodynamically unstable children, unresponsive to initial fluid therapy, cardiac output monitoring with echocardiography is recommended if available. Cardiac output could be monitored also with non-invasive or invasive pulse waveform analysis, although the data on the use of these methods in children are limited. Central venous pressure monitoring and central venous oxygen saturation monitoring could provide a more complex view of the patient’s status and its trend, but these types of monitoring should not be used as the sole method [69]. In children with pre-existing cardiac disease, transesophageal or transthoracic echocardiography could be helpful to determine the cardiac contractility, preload, signs of pulmonary hypertension, or a possible worsening of valvular diseases. The sources for this topic are rare, and the authors highlight the potential for further research in this area [9,36].

#### 3.3.4. Disability (Neurology, Analgosedation)

Dexmedetomidine sedation, in patients who require sedation after surgery, decreases the use of opioids and the risk of postoperative delirium when compared to midazolam [70].

Scoliosis correction is a type of surgery with an anticipated high postoperative pain level [70,71,72]. Patients with neuromuscular diseases are at a greater risk of undertreated pain [71,72]. Undertreated pain could lead to prolonged hospitalization, patient psychological trauma, and persistent postoperative pain [71]. The location of the most intense pain is the surgical wound. For most patients, it becomes tolerable after four days [71,72,73]. For this time period, the patient should be actively screened for their pain level and sufficiently treated. Multimodal analgesia should be chosen and started already during surgery to decrease opioid use. The most frequent opioid during surgery is remifentanil. Remifentanil’s conversion to longer-acting opioids, such as sufentanil or piritramide, is necessary before the end of the surgery. Postoperative continuous opioid administration by a patient-controlled analgesia (PCA) pump is the gold standard for patients after idiopathic scoliosis correction. Neuromuscular scoliosis is sometimes associated with syndromes characteristic of mental or severe physical impairments. Depending on the pain severity, the safe and effective use of PCA delivery may require the assistance of the pediatric patient’s parents and/or a nurse. Epidural analgesia and regional analgesia (bilateral erector spinae plane block) initiated at the operating theater reduce postoperative opioid use [71,72,73,74,75,76]. Epidural analgesia could achieve better analgesia than PCA. The patient’s neurologic status should be assessed before the administration bolus of local anesthetics because a sensory or motor blockade could occur. The risks associated with epidural analgesia do not differ from those of other surgery types [71,72]. For patients with idiopathic scoliosis, the intrathecal opioid (most often morphine or sufentanil) administration at the operation theater before the skin incision is associated with reduced intravenous opioid use during anesthesia and sufficient postoperative analgesia for the first 24 h. If morphine is lower than 20 µg/kg, the risk of respiratory depression is not higher than in the intravenous PCA method. Data on intrathecal morphine administration are limited in patients with neuromuscular scoliosis [71]. The bilateral erector spinae block in both the single-shot and catheter techniques has been successfully performed. The maximum dose of local anesthetic per 24 h needs to be respected to avoid systemic toxicity. Continuous wound infiltration seems to be an option for postoperative analgesia, but more research on this topic is required. Regional analgesia is often combined with non-steroidal anti-inflammatory drugs (NSAIDs) if needed [70,71,72,77]. Perioperative and postoperative low-dose ketamine infusion can also be an efficient part of multimodal pain management [77,78]. The intravenous use of local anesthetics (e.g., lidocaine) or gabapentinoids is controversial [70,77]. The indication, timing, and dosing scheme should undergo further research. The use of glucocorticoids should be explored more, but a single dose of dexamethasone after surgery seems to reduce the pain level without a higher risk of infection [70,77].

#### 3.3.5. Electrolytes

The syndrome of inappropriate antidiuretic hormone secretion (SIADH) can occur after spinal fusion in neuromuscular and idiopathic scoliosis. The therapy is not different compared to that for patients without neuromuscular disorders [79].

#### 3.3.6. Fluids (Kidneys)

Acute kidney injury (AKI) was detected in children after spine surgery. Different stages of AKI were diagnosed in 17% (35 of 208) of patients. The risk factors include nephrotoxic medications (e.g., aminoglycosides, NSAIDs) and a low amount of fluids intraoperatively. The management of NSAIDs is often needed in the postoperative period. However, their administration should be reconsidered daily because NSAIDs reduce prostaglandin synthesis. This can lead to kidney hypoperfusion or nephritis. Moreover, other nephrotoxic medication administration risks versus benefits should be reevaluated every day [80].

#### 3.3.7. Gastrointestinal Tract (GIT) and Nutrition

Preexisting dysphagia or gastroesophageal reflux can be worsened in patients with neuromuscular diseases. Tests and monitoring of dysphagia before postoperative realimentation can be helpful. Early postoperative nutrition should be started in malnourished patients prior to surgery. A good nutritional status can reduce the risk of postoperative infection [81]. Neuromuscular disease is also the leading risk factor for postoperative ileus, which is more likely in patients with extended bed rest. Monitoring symptoms of ileus (e.g., nausea, vomiting, abdomen distension) and early interventions can shorten the length of the hospital stay [81,82]. The risk of pancreatitis rises in cases of prolonged postoperative fasting. No cases of pancreatitis with organ failure, shock, or death have been reported. The impaired mental status or diminished pain due to high doses of analgesics and atypical clinical symptoms can make diagnostics difficult. Liver enzymes, amylase, and lipase measurement can help in obtaining a diagnosis. If pancreatitis develops, the standard treatment should be started immediately [82].

#### 3.3.8. Hematology

The more significant depletion of coagulation factors is present in patients suffering from neuromuscular disorders. Routine control of the thrombin time, prothrombin time, partial thromboplastin time, and fibrinogen in combination with viscoelastic hemostatic assays should be done in postoperative care. In prolonged postoperative bleeding, extended tests should be considered (e.g., factor XIII level), and the standard treatment for coagulopathy should be started [83].

#### 3.3.9. Infections

Infection is a leading complication in postoperative care after scoliosis surgery, with higher incidence in patients with neuromuscular disease. Urinary infection and pneumonia are the most frequent infections. The most common causes of urinary infections in hospitals are urinary catheters. Indications for the insertion and maintenance of urinary catheters should be reconsidered every day. Proper insertion techniques and maintenance care are a solution to reduce the risk of urinary infection [84]. The prevention of pneumonia is mostly the same as in other patients in the ICU. The specifics for patients with neuromuscular diseases are described above. Patients should also be routinely monitored for signs of wound infection [85,86]. The prevention of postoperative wound infection does not differ from standard care for wounds after spinal surgery.

#### 3.3.10. Rehabilitation

Patients with neuromuscular diseases are supposed to have complex physiotherapy. A very important part of rehabilitation is breathing and coughing exercises because of the high incidence of preoperative respiratory complications and weak cough. The pre-rehabilitation should start prior to the surgery to train high-risk patients to breathe with NIV or use a cough assistant. Chest physiotherapy should be maximized one week prior to surgery [24].

## 4. Conclusions

Anesthesia and the perioperative management of pediatric patients for neuromuscular scoliosis surgery represent a significant challenge for all healthcare providers. This heterogeneous team involves surgeons, anesthesiologists, neurophysiologists, pediatricians, nurses, nutritionists, and physiotherapists. Every part of the management should be adequately planned because of the higher risk of perioperative complications compared to idiopathic scoliosis. Healthcare providers have to consider all risks arising from the surgery, anesthesia, and the nature of the disease as a multidisciplinary and individual approach to each patient can improve the postoperative outcome. Firstly, mentioned data focus on the maximal optimization of altered functions before surgery and patients´ complex multidisciplinary prehabilitation. Secondly, adequate preparation of the anesthetic management regarding the specifics of neuromuscular syndromes, surgery, or anesthesia is essential for safety in the perioperative period. Thirdly, postoperative care in the ICU with adequate prevention, early identification, and treatment of possible complications can improve the postoperative outcome. All the described steps can lead to improved quality of life in patients with neuromuscular scoliosis.

## Data Availability

A narrative review with references retrieved from the databases of Embase, Google Scholar, Web of Knowledge, and PubMed was performed to obtain the related literature published.

## References

[1] El-Hawary R., Chukwunyerenwa C. (2014). Update on Evaluation and Treatment of Scoliosis. Pediatr. Clin. N. Am..

[2] Konieczny M.R., Senyurt H., Krauspe R. (2013). Epidemiology of adolescent idiopathic scoliosis. J. Child. Orthop..

[3] Sheehan D.D., Grayhack J. (2020). Pulmonary Implications of Pediatric Spinal Deformities. Pediatr. Clin. N. Am..

[4] Safari A., Parsaei H., Zamani A., Pourabbas B. (2019). A Semi-Automatic Algorithm for Estimating Cobb Angle. J. Biomed. Phys. Eng..

[5] Jin C., Wang S., Yang G., Li E., Liang Z. (2022). A Review of the Methods on Cobb Angle Measurements for Spinal Curvature. Sensors.

[6] Rüwald J.M., Eymael R.L., Upenieks J., Zhang L., Jacobs C., Pflugmacher R., Schildberg F.A. (2019). An Overview of the Current State of Pediatric Scoliosis Management. Z. Orthopädie Unf..

[7] Wishart B.D., Kivlehan E. (2021). Neuromuscular Scoliosis: When, Who, Why and Outcomes. Phys. Med. Rehabil. Clin. N. Am..

[8] Ridderbusch K., Spiro A.S., Kunkel P., Grolle B., Stücker R., Rupprecht M. (2018). Strategies for Treating Scoliosis in Early Childhood. Dtsch. Ärzteblatt Int..

[9] Halawi M.J., Lark R.K., Fitch R.D. (2015). Neuromuscular Scoliosis: Current Concepts. Orthopedics.

[10] Gibson P.R.J. (2004). Anaesthesia for Correction of Scoliosis in Children. Anaesth. Intensiv. Care.

[11] Weissmann K.A., Lafage V., Pitaque C.B., Lafage R., Huaiquilaf C.M., Ang B., Schulz R.G. (2020). Neuromuscular Scoliosis: Comorbidities and Complications. Asian Spine J..

[12] Google Scholar Google Scholar [Online]. https://scholar.google.com/.

[13] PubMed PubMed [online]. https://pubmed.ncbi.nlm.nih.gov/.

[14] Embase—A Biomedical Research Database. Elsevier | An Information Analytics Business [online]. https://www.elsevier.com/solutions/embase-biomedical-research.

[15] Web of Science. [Online]. https://www.webofscience.com/wos/author/search.

[16] Briggs E.D., Kirsch J.R. (2003). Anesthetic implications of neuromuscular disease. J. Anesth..

[17] Lerman J. (2011). Perioperative management of the paediatric patient with coexisting neuromuscular disease. Br. J. Anaesth..

[18] Turakhia P., Barrick B., Berman J. (2013). Patients with Neuromuscular Disorder. Med. Clin. N. Am..

[19] Thim T., Krarup N.H.V., Grove E.L., Rohde C.V., Løfgren B. (2012). Initial assessment and treatment with the Airway, Breathing, Circulation, Disability, Exposure (ABCDE) approach. Int. J. Gen. Med..

[20] Hudec J., Kosinova M. (2022). Anesthesia of the Patient with Zhu-Tokita-Takenouchi-Kim (ZTTK) Syndrome: A Case Report. Children.

[21] Sohn L., Peyton J., von Ungern-Sternberg B.S., Jagannathan N. (2021). Error traps in pediatric difficult airway management. Pediatr. Anesth..

[22] Hsu G., von Ungern-Sternberg B.S., Engelhardt T. (2021). Pediatric airway management. Curr. Opin. Anaesthesiol..

[23] Birnkrant D.J., Panitch H.B., Benditt J.O., Boitano L.J., Carter E.R., Cwik V.A., Finder J.D., Iannaccone S.T., Jacobson L.E., Kohn G.L. (2007). American College of Chest Physicians Consensus Statement on the Respiratory and Related Management of Patients With Duchenne Muscular Dystrophy Undergoing Anesthesia or Sedation. Chest.

[24] St-Laurent A., Zysman-Colman Z., Zielinski D. (2021). Respiratory prehabilitation in pediatric anesthesia in children with muscular and neurologic disease. Pediatr. Anesth..

[25] Racca F., Del Sorbo L., Capello E.C., Ranieri V.M. (2012). Neuromuscular patients as candidates for non invasive ventilation during the weaning process. Minerva Anestesiol..

[26] Katz J.A., Murphy G.S. (2017). Anesthetic consideration for neuromuscular diseases. Curr. Opin. Anaesthesiol..

[27] Racca F., Mongini T., Wolfler A., Vianello A., Cutrera R., Del Sorbo L., Capello E.C., Gregoretti C., Massa R., De Luca D. (2013). Recommendations for anesthesia and perioperative management of patients with neuromuscular disorders. Minerva Anestesiol..

[28] Pane M., Messina S., Bruno C., D’amico A., Villanova M., Brancalion B., Sivo S., Bianco F., Striano P., Battaglia D. (2013). Duchenne muscular dystrophy and epilepsy. Neuromuscul. Disord..

[29] Chou E., Lindeback R., Sampaio H., Farrar M.A. (2020). Nutritional practices in pediatric patients with neuromuscular disorders. Nutr. Rev..

[30] Wollinsky K.H., Weiss C., Gelowicz-Maurer M., Geiger P., Mehrkens H.H., Naumann T. (1996). Preoperative risk assessment of children with Duchenne muscular dystrophy and relevance for anesthesia and intra- and postoperative course. Med. Klin.—Intensiv. Und Notf..

[31] Vialle R., Thévenin-Lemoine C., Mary P. (2013). Neuromuscular scoliosis. Orthop. Traumatol. Surg. Res..

[32] Prottengeier J., Amann B., Münster T. (2020). Anästhesie bei neuromuskulären Erkrankungen. Anaesthesist.

[33] Roche D., Mahon P. (2021). Depth of Anesthesia Monitoring. Anesthesiol. Clin..

[34] Munshey F., Parra D., McDonnell C., Matava C. (2019). Ultrasound-guided techniques for peripheral intravenous placement in children with difficult venous access. Pediatr. Anesth..

[35] Keating G.M. (2016). Sugammadex: A Review of Neuromuscular Blockade Reversal. Drugs.

[36] Van de Voorde P., Turner N.M., Djakow J., de Lucas N., Martinez-Mejias A., Biarent D., Bingham R., Brissaud O., Hoffmann F., Johannesdottir G.B. (2021). European Resuscitation Council Guidelines 2021: Paediatric Life Support. Resuscitation.

[37] Tobias J.D. (2002). Controlled Hypotension in Children. Pediatr. Drugs.

[38] Schmitt H.J., Muenster T. (2008). Anesthesia in patients with neuromuscular disorders. Minerva Anestesiol..

[39] Glahn K.P.E., Ellis F.R., Halsall P.J., Müller C.R., Snoeck M.M.J., Urwyler A., Wappler F. (2010). Recognizing and managing a malignant hyperthermia crisis: Guidelines from the European Malignant Hyperthermia Group. Br. J. Anaesth..

[40] Gurnaney H., Brown A., Litman R.S. (2009). Malignant Hyperthermia and Muscular Dystrophies. Obstet. Anesth. Dig..

[41] Obata R., Yasumi Y., Suzuki A., Nakajima Y., Sato S. (1999). Rhabdomyolysis in association with Duchenne’s muscular dystrophy. Can. J. Anaesth..

[42] Gray R.M. (2017). Anesthesia-induced rhabdomyolysis or malignant hyperthermia: Is defining the crisis important?. Pediatr. Anesth..

[43] Sahinovic M.M., Gadella M.C., Shils J., Dulfer S.E., Drost G. (2021). Anesthesia and intraoperative neurophysiological spinal cord monitoring. Curr. Opin. Anaesthesiol..

[44] Yang J., Skaggs D.L., Chan P., Shah S.A., Vitale M.G., Neiss G., Feinberg N., Andras L.M. (2018). Raising Mean Arterial Pressure Alone Restores 20% of Intraoperative Neuromonitoring Losses. Spine.

[45] Kwee M.M., Ho Y.-H., Rozen W.M. (2015). The Prone Position During Surgery and its Complications: A Systematic Review and Evidence-Based Guidelines. Int. Surg..

[46] Grover M., Bachrach L.K. (2017). Osteoporosis in Children with Chronic Illnesses: Diagnosis, Monitoring, and Treatment. Curr. Osteoporos. Rep..

[47] Mirski M.A., Lele A.V., Fitzsimmons L., Toung T.J.K., Warltier D.C. (2007). Diagnosis and Treatment of Vascular Air Embolism. Anesthesiology.

[48] Reid J.M., Appleton P.J. (1999). A case of ventricular fibrillation in the prone position during back stabilisation surgery in a boy with Duchenne’s muscular dystrophy. Anaesthesia.

[49] Edler A., Murray D., Forbes R.B. (2003). Blood loss during posterior spinal fusion surgery in patients with neuromuscular disease: Is there an increased risk?. Pediatr. Anesth..

[50] Wang M., Zheng X.-F., Jiang L.-S. (2015). Efficacy and Safety of Antifibrinolytic Agents in Reducing Perioperative Blood Loss and Transfusion Requirements in Scoliosis Surgery: A Systematic Review and Meta-Analysis. PLoS ONE.

[51] Shapiro F., Zurakowski D., Sethna N.F. (2007). Tranexamic Acid Diminishes Intraoperative Blood Loss and Transfusion in Spinal Fusions for Duchenne Muscular Dystrophy Scoliosis. Spine.

[52] Esper S.A. (2011). Intra-operative cell salvage: A fresh look at the indications and contraindications. Blood Transfus..

[53] Klein A.A., Bailey C.R., Charlton A.J., Evans E., Guckian-Fisher M., McCrossan R., Nimmo A.F., Payne S., Shreeve K., Smith J. (2018). Association of Anaesthetists guidelines: Cell salvage for peri-operative blood conservation 2018. Anaesthesia.

[54] Sedra F., Shafafy R., Sadek A.-R., Aftab S., Montgomery A., Nadarajah R. (2020). Perioperative Optimization of Patients With Neuromuscular Disorders Undergoing Scoliosis Corrective Surgery: A Multidisciplinary Team Approach. Glob. Spine J..

[55] Koraki E., Stachtari C., Stergiouda Z., Stamatopoulou M., Gkiouliava A., Sifaki F., Chatzopoulos S., Trikoupi A. (2020). Blood and fluid management during scoliosis surgery: A single-center retrospective analysis. Eur. J. Orthop. Surg. Traumatol..

[56] Witmer C.M., Takemoto C.M. (2017). Pediatric Hospital Acquired Venous Thromboembolism. Front. Pediatr..

[57] Okamura M., Saito W., Miyagi M., Shirasawa E., Imura T., Nakazawa T., Mimura Y., Yokozeki Y., Kuroda A., Kawakubo A. (2021). Incidence of Unintentional Intraoperative Hypothermia in Pediatric Scoliosis Surgery and Associated Preoperative Risk Factors. Spine Surg. Relat. Res..

[58] Nemeth M., Miller C., Bräuer A. (2021). Perioperative Hypothermia in Children. Int. J. Environ. Res. Public Health.

[59] Brooks J.T., Yaszay B., Bartley C.E., Bastrom T.P., Sponseller P.D., Shah S.A., Samdani A., Cahill P.J., Miyanji F., Newton P.O. (2018). Do All Patients With Cerebral Palsy Require Postoperative Intensive Care Admission After Spinal Fusion?. Spine Deform..

[60] Akesen S. (2021). Predictive factors for postoperative ıntensive care unit admission in pediatric patients undergoing scoliosis correction surgery. Am. J. Transl. Res..

[61] Mills B., Bach J.R., Zhao C., Saporito L., Sabharwal S. (2013). Posterior Spinal Fusion in Children With Flaccid Neuromuscular Scoliosis. J. Pediatr. Orthop..

[62] Alexander W.M., Smith M., Freeman B.J.C., Sutherland L.M., Kennedy J.D., Cundy P.J. (2012). The effect of posterior spinal fusion on respiratory function in Duchenne muscular dystrophy. Eur. Spine J..

[63] Bach J.R., Sabharwal S. (2005). High Pulmonary Risk Scoliosis Surgery. J. Spinal Disord. Tech..

[64] Neto S.C.G.B., Torres-Castro R., Lima Í., Resqueti V.R., Fregonezi G.A.F. (2021). Weaning from mechanical ventilation in people with neuromuscular disease: A systematic review. BMJ Open.

[65] Hatef J., Hatef S., Drain J.P., Tobias J.D., Martin D., Shell R., Chase M., Beebe A., Samora W., Klamar J. (2022). Protocol-driven early tracheal extubation in patients with flaccid neuromuscular scoliosis and pre-existing lung disease. Spine Deform..

[66] Krishnakumar M., Muthuchellappan R., Chakrabarti D. (2020). Diaphragm Function Assessment During Spontaneous Breathing Trial in Patients with Neuromuscular Diseases. Neurocritical Care.

[67] Khirani S., Bersanini C., Aubertin G., Bachy M., Vialle R., Fauroux B. (2014). Non-invasive positive pressure ventilation to facilitate the post-operative respiratory outcome of spine surgery in neuromuscular children. Eur. Spine J..

[68] Levine S.B.B., Fields M.W., Boby A.Z.M., Matsumoto H.M., Skaggs K.F.B., Roye B.D.M., Vitale M.G.M. (2022). Degree of Postoperative Curve Correction Decreases Risks of Postoperative Pneumonia in Patients Undergoing Both Fusion and Growth-friendly Surgical Treatment of Neuromuscular Scoliosis. J. Pediatr. Orthop..

[69] Singh Y., Villaescusa J.U., da Cruz E.M., Tibby S.M., Bottari G., Saxena R., Guillén M., Herce J.L., Di Nardo M., Cecchetti C. (2020). Recommendations for hemodynamic monitoring for critically ill children—Expert consensus statement issued by the cardiovascular dynamics section of the European Society of Paediatric and Neonatal Intensive Care (ESPNIC). Crit. Care.

[70] Aydogan M.S., Korkmaz M., Ozgül U., Erdogan M.A., Yucel A., Karaman A., Togal T., Durmus M., Colak C. (2013). Pain, fentanyl consumption, and delirium in adolescents after scoliosis surgery: Dexmedetomidine vs. midazolam. Pediatr. Anesth..

[71] Seki H., Ideno S., Ishihara T., Watanabe K., Matsumoto M., Morisaki H. (2018). Postoperative pain management in patients undergoing posterior spinal fusion for adolescent idiopathic scoliosis: A narrative review. Scoliosis.

[72] Dinter K., Bretschneider H., Zwingenberger S., Disch A., Osmers A., Vicent O., Thielemann F., Seifert J., Bernstein P. (2021). Accelerate postoperative management after scoliosis surgery in healthy and impaired children: Intravenous opioid therapy versus epidural therapy. Arch. Orthop. Trauma Surg..

[73] Chiu C.K., I Chong K., Chan T.S., Mohamad S.M., Hasan M.S., Chan C.Y.W., Kwan M.K. (2020). The anatomical locations of postoperative pain and their recovery trajectories following Posterior Spinal Fusion (PSF) surgery in Adolescent Idiopathic Scoliosis (AIS) patients. Med. J. Malaysia.

[74] Chin K.J., Lewis S. (2019). Opioid-free Analgesia for Posterior Spinal Fusion Surgery Using Erector Spinae Plane (ESP) Blocks in a Multimodal Anesthetic Regimen. Spine.

[75] Shrader M.W., Nabar S.J., Jones J.S., Falk M., Cotugno R., White G.R., Segal L.S. (2015). Adjunctive Pain Control Methods Lower Narcotic Use and Pain Scores for Patients With Adolescent Idiopathic Scoliosis Undergoing Posterior Spinal Fusion. Spine Deform..

[76] Diwan S., Altinpulluk E.Y., Khurjekar K., Nair A., Dongre H., Turan A. (2020). Bloqueo bilateral en el plano del músculo erector de la columna para cirugía de escoliosis: Serie de casos. Rev. Española Anestesiol. Reanim..

[77] Lee C.S., Merchant S., Chidambaran V. (2020). Postoperative Pain Management in Pediatric Spinal Fusion Surgery for Idiopathic Scoliosis. Pediatr. Drugs.

[78] Zhou L., Yang H., Hai Y., Cheng Y. (2022). Perioperative Low-Dose Ketamine for Postoperative Pain Management in Spine Surgery: A Systematic Review and Meta-Analysis of Randomized Controlled Trials. Pain Res. Manag..

[79] Lieh-Lai M.W., Stanitski D.F., Sarnaik A.P., Uy H.G., Rossi N.F., Simpson P.M., Stanitski C.L. (1999). Syndrome of inappropriate antidiuretic hormone secretion in children following spinal fusion. Crit. Care Med..

[80] Jöbsis J.J., Alabbas A., Milner R., Reilly C., Mulpuri K., Mammen C. (2017). Acute kidney injury following spinal instrumentation surgery in children. World J. Nephrol..

[81] Verhofste B.P., Berry J.G., Miller P.E., Crofton C.N., Garrity B.M., Fletcher N.D., Marks M.C., Shah S.A., Newton P.O., Harms Study Group (2020). Risk factors for gastrointestinal complications after spinal fusion in children with cerebral palsy. Spine Deform..

[82] Bureta C., Tominaga H., Yamamoto T., Kawamura I., Abematsu M., Yone K., Komiya S. (2018). Risk Factors for Postoperative Ileus after Scoliosis Surgery. Spine Surg. Relat. Res..

[83] Kannan S., Meert K.L., Mooney J.F., Hillman-Wiseman C., Warrier I. (2002). Bleeding and coagulation changes during spinal fusion surgery: A comparison of neuromuscular and idiopathic scoliosis patients. Pediatr. Crit. Care Med..

[84] Gould C.V., Umscheid C.A., Agarwal R.K., Kuntz G., Pegues D.A. (2010). Healthcare Infection Control Practices Advisory Committee (HICPAC) Guideline for Prevention of Catheter-Associated Urinary Tract Infections 2009. Infect. Control. Hosp. Epidemiol..

[85] Yousef M.A.A., Rosenfeld S. (2018). Evaluation of postoperative fever after surgical correction of neuromuscular scoliosis: Implication on management. Eur. Spine J..

[86] Shillingford J.N., Laratta J.L., Reddy H., Ha A., Lehman R.A., Lenke L.G., Fischer C.R. (2018). Postoperative Surgical Site Infection After Spine Surgery: An Update From the Scoliosis Research Society (SRS) Morbidity and Mortality Database*. Spine Deform..

